# Dr. Blegborough, on Animal Heat in Typhus, &c.

**Published:** 1804-09-01

**Authors:** Ralph Blegborough

**Affiliations:** Margaret Street


### *To the Editors of the Medical and Physical Journal.*

"His Highness yet doth speak; and holds belief,

"That being brought into the open air,

"It would allay the burning quality

"Of that fell poison, which assaileth him."

King John, Act 5, Sc. 7.

Gentlemen,

After having just now, for the first time, finished the perusal of Dr. Currie's valuable Reports on the Effects of Water in Fever, I sit down to communicate a few observations which have occurred to me on the subject.

I do it with the greater confidence, as that good man and great physician has done me the honour to mention with respect a communication of mine to your Journal for August, 1802; a communication which arose out of the painful personal feelings of my own case, and that of others of my family, which did not fail to awaken every energy of my mind. On that occasion, I expressed an idea, which had taken strong hold of my imagination, that as the discharge of gonorrhœa is suspended by an astringent injection, so typhus fever might be by affusion; but that some specific disposition of generating preternatural heat, remained in the system for fourteen days. This no doubt was a relic of the old and ridiculous critical-day system, and I am very happy to sacrifice it at the shrine of truth, established by Dr. Currie's more bold and decisive practice. I cannot, however, help flattering myself with the idea, that, though I may in some instances have unnecessarily encroached on the time of my patients, I do not recollect one case in which the plan eventually failed me; and that in all, I avoided the risk of syncope, which it appears has happened to some of Dr. Currie's communicants; and the probable occurrence of which, from the too sudden abstraction of heat, I trust I shall be able to elucidate before I bring this paper to a close.

With regard to the theory of fever, I am perfectly satisfied with the modification which Dr. Currie has given to that of Dr. Cullen; and think it the best that can be given in the present imperfect state of our knowledge respecting the laws of animal life. I have always been of opinion, that there was no occasion for *synocha* in our discrimination of fevers, and have seldom been at a loss to account for for every excitement of the system, which did not ultimately terminate in typhus. In this disease, owing to the spasm on the minute vessels on the surface of the body, these vessels, and the skin itself, become bad conductors of heat. This subtile elastic fluid, which during a free circulation is carried along with the blood to the extreme vessels, and is constantly passing off in perspiration, is, under these circumstances, accumulated in the centre of the body in the large vessels, distending them in such a manner, as to render them incapable of contracting so as to propel it to the surface; just as we find the bladder once distended much beyond its tone with urine, incapable any longer of contracting to expel its contents.

The suffusion of the eye, however, shews distinctly not only the distensive, but also the stimulating property of the matter of heat, where this spasm and resistance do not exist, at least to the same degree.

The idea that heat is a material substance, and may be pressed out of bodies like water out of a sponge, is a beautiful and correct comparison, and if kept in view, may be made very subservient, I apprehend, to a better conception of the nature, not only of typhus fever, but of all diseases of increased action of the vascular system. Your readers do not need to be informed of the common expansive power of heat; nor that a certain quantity of some elastic fluid (for philosophers I observe have been rather shy of giving it a specific name) is diffused through every part of the human body, to counteract the pressure of the external atmosphere.

Now, when we consider that the capacity of the bones of the head is but just sufficient to contain the brain; and that the most trifling thing insinuated between the bones of the head and brain, is not only capable of disturbing the functions of that organ, but not unfrequently of occasioning even the death of the patient; we shall not be surprized, if an increased quantity of heat, circulating along with, and distending the fluids, should produce exactly the same effects. If I mistake not, Sir George Shuckburgh has proved the expansion even of common air to increase at the rate of the 440th part of its volume, for each degree of increased heat on the scale of Fahrenheit. The circumstance of this expansion is easily proved by holding a bladder partly inflated before the fire; for no sooner is the included air affected by the heat, than it begins to expand itself and distend the bladder.

Now, I suppose, it is generally known that the increase of temperature in typhus fever, and other diseases of increased excitement, is not unfrequently 8 or 10, and sometimes more, degrees higher than the ordinary temperature of the human body. This expansion, increasing in a compound ratio, according to the above reasoning, must not only greatly distend the volume of the fluids circulating through the vessels of the brain, but must also at the same time much diminish the internal area of the vessels themselves, and of course their capability of freely circulating such fluids. The bones of the head will not be proportionably, if at all, dilated; and of course must resist the external dilatation of the vessels contained within them. The obvious consequence must be (if I may so phrase it) a *congeries of compression.* And I have little doubt, that while this account of the matter accords with the soundest principles of philosophy, it applies well also to the general circumstances which take place in typhus, and accounts for the disturbed functions of the brain in this disease; the cure of which is most effectually accomplished, as Dr. Currie and other philosophical practitioners have abundantly proved, by reducing the temperature, or, in other words, by abstracting the matter of heat from the system. The pain which takes place in the breast and back, may also be accounted for on the same principle.

Dr. Gregory, my much respected teacher, who has completely anticipated the remarks I had to offer on *scarlatina*, when on the subject, *De Sanguinis naturâ*, in his valuable *Conspectus*, says, "*Sanguis e venis missus, tenuem vaporem primo exhalat, fere aquosum, levissime olidum, sed parcâ admodum quantitate.*" Now, though the quantity of this vapour in the blood may be small, can we be at a loss to account for the power of this active agent, heat, upon it, in producing the above consequences? or hesitate a moment respecting the propriety of abstracting part of it, when so morbidly increased, from the system, by the best means we are able to employ? when we consider that the particles of a single drop of water, may be distended so as by its elastic power to raise a ton weight, or the mischief it is capable of producing, if accidently dropped into the mould, in which a cannon is about to be cast; when we consider the above experiment of Sir George Shuckburgh, and how much more expansible this vapour may be than common air, and that the temperature of the human body, under particular circumstances, varies from 84 to 112, as we learn from Dr. Currie's Reports. From considering the matter in this point of view, I apprehend we shall no longer longer be at a loss to account for the exacerbations and remissions of fever; the first taking place generally about five o'clock in the afternoon, the time immediately succeeding the hottest part of the day; the latter about the same hour in the morning, generally the coolest part of the night.

As syncope frequently occurs from too suddenly evacuating the contents of the abdomen in ascites, so I apprehend it will frequently follow the too sudden abstraction of heat in typhus; and thus, instead of abstracting it more slowly, and giving the vessels an opportunity to contract gradually, by the sudden dash we may instantly so diminish the volume of their contents, that they may not be able to contract upon them. This seemed to happen in the case of Dr. Ord, and appears to render the opinions of Dr. Jackson and Dr. M'Lean, that it acts only through the medium of sensation, very untenable indeed. Dr. Macneil seems also to think the sponging is better than the dash. The more this subject, in my opinion, is considered in a mechanical point of view, the sooner will correct ideas be formed respecting it: On this account I shall merely allude to the circumstance of the sudden collapse of vapour, in the piston of a steam engine, from a jet of cold water. I am confidently assured, that it is no unusual sight in the West Indies to see pigs, which have been exposed to excessive heat in being brought to the towns in baskets, on the heads of negroes, instantly die on being affused with cold water. The small space which the blood seems to occupy in animals that have been frozen to death, is also illustrative of this circumstance.

The sudden and unequal contraction of the stomach, from taking too great a quantity of cold liquid into it at once, has frequently been known to occasion unpleasant consequences. I experienced myself the most extreme pain about a fortnight ago, from this circumstance. By a due consideration of the subject in this point of view, and that the temperature of the human body may be made to vary, as has been shewn, near thirty degrees, few I apprehend will doubt, that a bulk of material substance may thus be abstracted from the system, equal to, if not greater than, six or eight times the quantity of blood that could in any case with safety be taken away. I also think it highly probable, that such a quantity of the matter of heat will be no less stimulating than an equal bulk of blood would be, provided the temperature of the body was diminished in an adequate proportion. I always

R 4 counteract counteract the temporary excitement consequent on good fellowship by almost constantly sponging myself with cold water till it ceases; and was much amused with Mr. Weekes's plan, as stated by Dr. Currie.

I own I am an advocate for the mechanical theory of the production of animal heat, and think this manner of accounting for it has evidently the advantage of the present chemical one. There are insuperable objections, I think, to that of the ingenious Mr. Rigby, that animal heat is generated by the decomposition of the food in the stomach and bowels. For the complete refutation of latent heat and all its dependences, see my ingenious friend Mr. Tilloch's account of the matter in his Philosophical Magazine for 1800. By the bye, I was much surprized not to find the name of Rigby, who has done so much for the philosophy of medicine, not only in this but in other departments of it, once occur in the whole of Dr. Currie's work; which makes me conclude that Dr. C. cannot have read his book on Animal Heat. Throughout that whole work, Mr. Rigby, with the true spirit of a philosopher, regrets that from extensive practice his opportunities of acquiring philosophical knowledge, had not been so great as he could have wished; and seems fully sensible that those improvements in our profession, which are established on natural knowledge, are always the most durable and most easily comprehended. Such, I trust, the plan, so fully, explicitly, and ingenuously treated by Dr. Currie, will appear; and I think he need not be very solicitous about its fate; as from its merits it cannot be long before it be generally adopted, "*even in London.*"

A friend of mine, an eminent physician, lately had the disease here. He was attended by three physicians of great celebrity. It appeared he had every bad symptom, and was in imminent danger; when his lady, a woman of a strong mind, with more affection for her husband than regard for the dogmas of the schools, ventured on the cold bathing plan, which happily was in time to recover the patient. I forbear to mention the name, as I have not had an opportunity before I send this of asking leave to do so: the name, however, would add dignity to this communication. I own it would afford me great pleasure to see the advocates of the old system adopt the one inculcated here, or give some good reason for not doing it. I wish much to have our profession considered not as a trade, but as a science, connected with other branches of philosophy, and its principles reasoned on accordingly.

The

The yellow fever of the West Indies, and the plague itself, I should apprehend, are but modifications of typhus; their excessive malignancy depending on the more quick and rapid excitation of heat: I speak this, however, with that diffidence which ought to belong to one who has never seen either disease. Dr. Falconer, it seems, thinks the latter may be prevented by cold bathing. If I should differ in opinion from this most respectable physician, I should not hesitate a moment to think myself in the wrong; but really I cannot see why both diseases may not only be prevented, but cured also, by a judicious and scientific administration of the plan.

I am aware that some of these suggestions will appear, to many of your readers, altogether Utopian. I care not, however, if I make but one proselyte. Regardless about the fate of the theory, I am desirous only of establishing the facts; and it is my intention to prosecute this subject more in detail on some future occasion.

I have seen cases similar to that mentioned in page 47 of Dr. Currie's work, and ask, what effects might be expected in such cases, from occasionally sponging the head with brandy or even æther, while the rest of the body was kept cool by affusion?

I have long been in the habit of estimating heat as a mechanical power, and acting with it accordingly in the treatment of disease; nor have I, under any circumstances, found reason to doubt of my theory. Nothing more is necessary in my opinion to establish Dr. Currie's views, than strongly to inculcate this idea.

Convinced of their truth and importance, may his Medical Reports find many Echoes on *this* side of our happy Island; and may he long continue to enjoy the satisfaction, which cannot fail to result from the success of his well-conducted labors.

*Margaret Street,* I am, &c.

*Aug.* 6, 1804. RALPH BLEGBOROUGH.

